# eHealth in the future of medications management: personalisation, monitoring and adherence

**DOI:** 10.1186/s12916-017-0838-0

**Published:** 2017-04-05

**Authors:** Josip Car, Woan Shin Tan, Zhilian Huang, Peter Sloot, Bryony Dean Franklin

**Affiliations:** 1 Centre for Population Health Sciences, Lee Kong Chian School of Medicine, Nanyang Technological University, 3 Fusionopolis Link, #06-13, Nexus@One-North, South tower, Singapore, 138543 Singapore; 2 Global eHealth Unit, Department of Primary Care and Public Health, School of Public Health, Imperial College, London, UK; 3 Nanyang Institute of Technology in Health and Medicine, Interdisciplinary Graduate School, Nanyang Technological University, Singapore, Singapore; 4 Health Services and Outcomes Research Department, National Healthcare Group, Singapore, Singapore; 5 Computational Science Laboratory, University of Amsterdam, Amsterdam, The Netherlands; 6 ITMO University, Saint Petersburg, Russia; 7 Complexity Institute, Nanyang Technological University, Singapore, Singapore; 8 Research Department of Practice and Policy, UCL School of Pharmacy, London, UK; 9 Centre for Medication Safety and Service Quality, Pharmacy Department, Imperial College Healthcare NHS Trust, London, UK

**Keywords:** Apps, eHealth, mHealth, Drug monitoring, Information communication technology, Medication adherence, Medication therapy management, Text message

## Abstract

**Background:**

Globally, healthcare systems face major challenges with medicines management and medication adherence. Medication adherence determines medication effectiveness and can be the single most effective intervention for improving health outcomes. In anticipation of growth in eHealth interventions worldwide, we explore the role of eHealth in the patients’ medicines management journey in primary care, focusing on personalisation and intelligent monitoring for greater adherence.

**Discussion:**

eHealth offers opportunities to transform every step of the patient’s medicines management journey. From booking appointments, consultation with a healthcare professional, decision-making, medication dispensing, carer support, information acquisition and monitoring, to learning about medicines and their management in daily life. It has the potential to support personalisation and monitoring and thus lead to better adherence. For some of these dimensions, such as supporting decision-making and providing reminders and prompts, evidence is stronger, but for many others more rigorous research is urgently needed.

**Conclusions:**

Given the potential benefits and barriers to eHealth in medicines management, a fine balance needs to be established between evidence-based integration of technologies and constructive experimentation that could lead to a game-changing breakthrough. A concerted, transdisciplinary approach adapted to different contexts, including low- and middle-income contries is required to realise the benefits of eHealth at scale.

## Background

Up to half of medicines prescribed for long-term conditions are not taken as recommended [1, 2]. An estimated US$375 billion per year could be saved with improved medication adherence [3], which could prove to be one of the most effective interventions for improving health outcomes [4, 5]. Reasons for non-optimal use of medicines are multifactorial and may be related to patient, therapy, disease or health system issues [6]. Numerous studies have aimed to improve medication adherence by addressing factors such as health literacy, lack of motivation or understanding, trust in the physician, health behaviour, cognitive factors such as forgetfulness, and others [7–10].

With regards to the patient, barriers to effective medicines management in chronic illnesses are multifactorial; medication adherence can be intentional or unintentional [11–14]. Unintentional non-adherence arises due to several inter-related factors, including inability to keep up with medication dosing schedules, long duration and complexity of medication regimens, miscommunication, and capacity limitations such as physical disabilities and financial constraints [11, 14]. Intentional non-adherence is a more complex problem and involves patients choosing not to take their medications as prescribed due to various behavioural, attitudinal and socioeconomic reasons [11, 15]. Several interventions have shown to improve medication adherence [16], but most at best by only 10 percentage points [16, 17]. Very few interventions have been implemented at a large scale as these interventions are typically complex, labour intensive and costly [18].

**Fig. 1:**
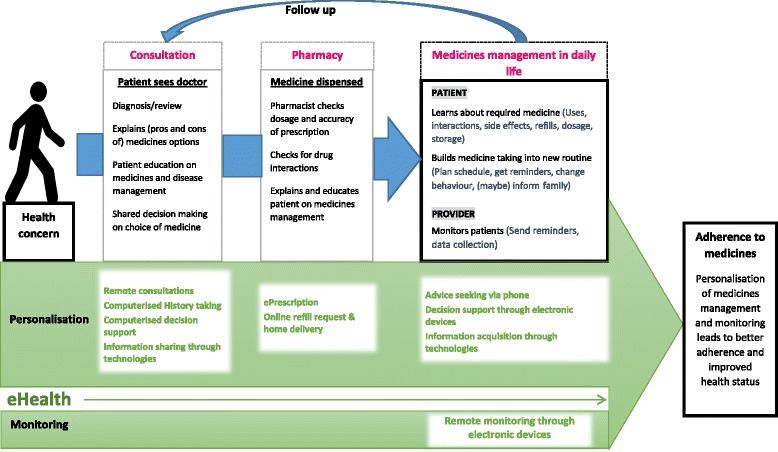
Role of eHealth in enhancing personalisation and monitoring in patients’ medicines management journey

We conducted a literature review on topics related to eHealth and medicines management on PubMed. Conference proceedings and national guidelines were also searched. This paper is not a systematic review of literature, but a summarised discussion of eHealth in the patients’ medicines management journey. In this discussion, we explore the role that eHealth (the use of a wide range of information and communication technologies for health) (Table 1) could play in primary care, outpatient and community medicines management, focusing on how more personalisation and more intelligent monitoring could lead to greater adherence (Fig. 1).

**Table 1:** eHealth technologies and their application in medicines management

eHealth technology	What is it?	Application in medicines management
Electronic health records (EHRs)	EHRs are real-time, patient-centred records that provide immediate and secure information to authorised users. EHRs typically contain a patient’s medical history, diagnoses and treatment, medications, allergies, immunisations, as well as radiology images and laboratory results. A national EHR system is most-often implemented under the responsibility of a national health organisation and will typically make a patient’s medical history available to health professionals in healthcare institutions and provide linkages to related services such as pharmacies, laboratories, specialists, and emergency and medical imaging facilities [62]	History taking, recording and decision support for medicines management
Telehealth	Telehealth refers to the delivery of healthcare services, where patients and providers are separated by distance. Telehealth uses information communication technologies for the exchange of information for the diagnosis and treatment of diseases and injuries, research and evaluation, and for the continuing education of health professionals. It is particularly valuable for those in remote areas, vulnerable groups and aging populations [63]	Remote consultations including exchange of information on chronic disease care and medicines management [64]
Web-based monitoring system	Web-based monitoring systems are internet-based websites developed to monitor and/or to deliver tailored educational content related to a health condition	Monitoring of medication adherence and education on the chronic disease, including the required medication (e.g. asthma) [65]. May/may not provide a communication platform for patients with their healthcare providers [39]
Short messaging service (SMS)	SMS, or text messaging, is used as a communication tool to exchange short messages over the phone, web or mobile communication system. It is a low-cost method to promote, inform or remind patients on information relating to their health or healthcare. It can also be used as a tool to facilitate communication of health messages between patients and healthcare providers [36]	Tailored or standardised medicine taking reminders with either one way or two-way interactions. Studies have shown improvement in medication adherence, but more high quality studies are required to establish evidence in this area [66]
Mobile health apps	Mobile apps are software applications designed to run on portable devices such as smartphone or tablet computers. Health apps are designed for a multitude of purposes such as data collection, health and disease education, disease and lifestyle management, surveillance, monitoring and health promotion to support the management of one’s health	Personalised reminders; medicines organisation; polypharmacy management; integration of medicines taking as part of chronic disease management; information exchange on medicines; medication refill orders
Wearable devices	Wearable devices are gadgets embedded with electronics, software or sensors that can be worn as clothing or accessories. Wearable devices are also examples of Internet of Things, where a network of sensors can perform data exchange across platforms. These devices are useful for round-the-clock monitoring and can collect real-time data through location and activity tracking	App-linked devices with sensors can be worn to alert asthma patients of impending asthma attacks, for better medicines management [67]. Biosensors such as wearable patches or contact lenses can continuously measure blood glucose for better insulin management [68]; however, the efficacy and cost-effectiveness of these devices have yet to be established

## The patient’s medicines management journey

The medicines management process usually starts when the patient first gains access to a healthcare provider, and jointly decides with the prescriber on the prescription during a clinical consultation. After obtaining the medicines at a pharmacy, the patient must integrate the process of medication taking into their daily routine. Non-adherence can occur when the patient does not take the first dose, does not continue with the prescribed treatment at the approriate frequency or dose, or does not continue taking the medicines after a period of time [19].

### Access to healthcare providers

eHealth is changing conventions for how patients consult their health providers. For an ever growing number of scenarios, face-to-face consultations that were once the norm are being supplemented or replaced with a range of synchronous or asynchronous remote consulting approaches via text, voice or video. For example, it might be suggested that the review of medication for patients with a chronic illness that does not require physical examination can be done remotely, patients with concerns about accidental overdosing can phone for advice, and less urgent queries can be made through an email or web portal. Remote consulting does not just improve convenience and access, save travel and waiting time, but also removes geographical distances and potentially reduces socioeconomic gaps.

Automated telephone communication systems (ATCS) can deliver pre-recorded voice messages from healthcare providers to patients through the telephone’s touch-tone keypad or via voice-recognition software. ATCS have been shown to provide remote, round-the-clock access to healthcare advice for patients with lower socio-economic backgrounds, and are able to facilitate self-monitoring in various chronic diseases such as diabetes, heart failure and hypertension, although the evidence is mixed [20].

Appointment scheduling and reminders for medication reviews can equally benefit from these technologies [21, 22]. The key challenge is to provide seamless integration into health systems to enable discrete, yet effective communication of prompts and reminders, and increase flexibility to change scheduled appointments effortlessly if required. Few health systems have achieved this at scale and made it routine care. Yet, both in terms of evidence and technology, there are few barriers to make it happen other than the redesign of older, less effective, processes. This should be realised, as with all (e)health interventions, within the framework of continuous evaluation to ensure further improvement and learning.

The options for eHealth technologies are expected to further expand to allow more flexibility in accessing healthcare. Smartphones are increasingly all-pervading healthcare tools, able to offer health advice at just a ‘tap’ away. Medicines can also be ordered through a smartphone app and delivered to patients’ homes. Moving forward, there is a need to establish evidence for the right balance in use of these novel technologies. Outcomes, such as quality and safety of care, cost-effectiveness, acceptability, fidelity, etc., all need to be rigorously evaluated in order to understand how best to reap the potential benefits while minimising potential limitations.

### Clinical consultations

When patients enter the privacy and uniqueness of human trust in a medical consultation, what follows is a careful history taking, examination and investigation, diagnosis and review, as needed. Further to making a diagnosis or review, the physician or other healthcare professional will explain to the patient their treatment options and discuss the disease and medicines management. In the past, patients were expected to follow doctors’ choices and recommendations, including those relating to medicines, without active involvement in decision-making. Today, it is recognised that personalising healthcare within a shared decision-making framework leads to better outcomes, patient engagement and increases the level of trust between providers and patients [23].

eHealth is able to improve pre-consultation medical history taking by digitising information acquisition for history taking via Computer Assisted History Taking Systems (CAHTS). The use of CAHTS has the potential to improve quality and comprehensiveness of information captured, reduce data entry errors, save time and facilitate acquisition of sensitive patient information [24]. It can be used from the convenience of home, while travelling, or while in the waiting room. CAHTS can collect information on medication history, adherence and possible side effects, and can use decision support trees in data capture, etc. It is more effective than face-to-face consultation in capturing sensitive information (e.g. about sexually transmitted diseases, or mental illness). Use of CAHTS will increase in the coming years as it advances in sophistication and becomes more routinely adopted within healthcare.

eHealth is transforming decision-making, information sharing and patient education. Within medication management, electronic health records (EHRs), computerised prescription order entry (CPOE) and computerised decision support systems (CDSS) are now considered the norm in many health systems, albeit with scope for further development [25]. For example, CDSS can improve patient safety through checks for drug allergies and drug-drug interactions, and by providing evidence-based recommendations and prescribing guidelines. Direct transmission of prescriptions to the pharmacy can also reduce transcription errors. Relevant information on the individual’s current and past prescriptions, or medicines taking behaviour, can support personalisation of care by informing providers about potential non-adherence and contraindicated therapies. Patient education and medicines management plans can then be tailored to focus on individual needs, which is more likely to support adherence to planned treatment.

### Dispensing of medication

Similar to the flight attendants’ pre-flight cross-checking of an aircraft’s doors, the pharmacy acts as a cross-check for the prescriber by ensuring the medications prescribed are safe in terms of dose, frequency and the presence of other medications, and appropriate for the patient according to their illness and medication history. In the future, we anticipate that eHealth could support and further this cross-checking function. Prior to dispensing the medication at the pharmacy, using interactive technology, eHealth solutions could check for patients’ understanding of medications based on what was conveyed by the doctor during the consultation. This could support patients’ adherence through better patient education.

ePrescription potentially represents digitisation of both prescribing and dispensing. With increasing automation of dispensing, this has the potential to free up pharmacists’ time to spend in clinical roles such as talking to patients to support adherence. The role of the pharmacist is increasingly shifting from medication dispensing to the automation of pharmacy dispensing functions [26]. In addition to the ability to reduce transcription errors through electronic transmission of orders from the prescriber to the pharmacy, ePrescribing solutions can also support medication reconciliation, remote monitoring of adherence through refill tracking, and issuing of automatic refill alerts [27]. Patients can also benefit from a seamless medication refill process and the convenience of home delivery of medications. In many high-income countries, ePrescription has transformed prescribing and dispensing with automated decision support. Health systems that allow prescribers and pharmacists to access the information pertaining to an individual’s prescriptions, and support the prescription of formulary alternatives such as generic drugs, could lower the cost to patients and healthcare systems. Although the technology is mature, adoption among solo or small primary care practices is still low in third-party payer systems such as the USA [28]. Being able to expand coverage in the future to span the entire health economy will also prevent medication safety issues that arise as a result of care fragmentation. On the other hand, some countries do not have healthcare organisations that are supported by an interoperable information technology network. Although CPOE systems can be standalone, the issue of fragmentation of medication records could act as a barrier towards reaping the full benefits of an integrated IT system. This could change in the future with greater integration of IT solutions within healthcare.

In the future, we might expect complete digitisation and the integration of CDSS and CPOE with personal health records and medical records to provide a more seamless patient experience. Being able to interact with the healthcare system via online personal health portals has been shown to improve patients’ medication adherence [29] due to the added convenience of online medication refill and also through increased awareness of their health status [30]. Due to the impact on revenue and patient outcomes, the pharmaceutical industry, as well as public and private funders, are ardently exploring eHealth technologies to improve medicines management. A number of pharmaceutical companies have launched apps for medication adherence and chronic disease medicines management, and moving towards more partnerships with healthcare institutions to improve the quality and delivery of eHealth technologies.

At the system level, with greater information integration, a self-learning IT system could be created. For example, new information about the individual patient, especially health behaviour data that might be captured via wearables, can offer better identification of individuals who need additional support or change of treatment.

## Medicines management in daily life

Self-management and medicines taking occurs after the patient leaves the pharmacy with the dispensed medicine. Newly diagnosed patients learn about the medicine (e.g. about its uses, interactions, precautions, side effects, dosage, storage) and incorporate, with varying degrees of success, their medicine taking into daily life routines at home, work or while travelling.

Apart from medicines management education received in consultation with the prescriber and the pharmacist, patients may have subsequent questions about the medicines they are taking. The internet and multimedia-rich mobile phone applications (apps) already act as information sources and will increasingly do so, potentially improving health literacy by providing and reinforcing education on topics such as the purpose of the medicine, how to take it, possible side-effects and contraindications.

To reinforce adherent behaviours, electronic reminders can alert patients to take their medication, to monitor parameters, or about upcoming appointments. Tailoring of the frequency and timing of mobile phone text messages is increasingly being used to remind patients of their medicines schedule [31]. Tailored interventions have also shown superior outcomes in behavioural change compared to standardised text messages [32]. Text messages in studies showing a benefit were more likely to contain educational and motivational content and/or were more tailored to the user or their condition. More basic and repetitive text messages showed fewer benefits [33]. Personalised interventions based on shared decision-making with patients are more likely to have positive outcomes, as involving patients in deciding the time and method for receiving interventions from healthcare providers minimises annoyance from constant notification of health messages [34]. In addition, studies have found that receptivity towards mobile phone text messages as a healthcare intervention reduces with increasing age, as well as with lower education and income levels [35]. Therefore, clinicians and policymakers need to be cognizant of this and provide additional strategies for certain patient groups.

Monitoring of patient outcomes and medicines taking behaviour are important functions to achieve better adherence to medicines. A survey by the WHO on mobile health (mHealth) reported the highest percentage of patient monitoring activities in high-income countries compared to other world regions but patient monitoring emerged as the least established mHealth initiative despite its strong potential for improving outcomes and cutting healthcare costs [36]. A systematic review also showed a reduction in all-cause mortality and hospital admission by approximately 20% in patients with chronic heart failure undergoing remote monitoring compared to patients under usual care [37]. The process of medicines taking can be self-monitored or performed by the primary care provider using electronic devices. To enable personalised self-care, smart devices, consumer wearables, electronic pill boxes and applications (device agnostic, mobile or other) should be designed to be easy to use with customisable features. These electronic devices could then concurrently support the goal of information gathering for healthcare decision support.

eHealth advances will continue to drive rapid progress to close the current gaps within the medicines management process by enhancing the speed and accuracy of data transmission. The Internet of Things (IoT) is a concept referring to networked everyday objects that interconnect to each other via wireless sensors attached to them [38]. With the emergence of IoT, information on consultations, prescriptions and medication refills can be integrated into a centralised system combined with a new previously non-existent category of personally collected data about patients, which will allow for a new level of insight into patients’ progress and new level of personalisation of treatment. Internet interventions and short messaging service (SMS) reminders have shown promising results in enhancing patients’ adherence to long-term medications [39–41].

## Support for carers

The role of carers has grown in importance as support from friends and family members is a key facilitator of medication adherence [11]. Many carers are unprepared for the demands of caring for a chronically ill family member and will require support and training in the process of caring for their loved ones. To date, eHealth has shown moderate effects, for example, on improving carer stress and depression in caring for people with dementia [42]. Carers of patients with polypharmacy will likely benefit from peer support, medicine reminders, organisation, information exchange on medicines and digital encouragement with the support of eHealth.

## Data analytics

The growing trend of ubiquitous continuous monitoring via mobile phones, wearables, smart pill boxes, IoT, etc., present an opportunity to gain a new level of insight into a wide range of parameters which directly or indirectly affect health outcomes. Combined with the data gathered by the health system for an individual as well as whole populations at the level of the clinic, pharmacy, hospitals, etc., new predictive models and stratification approaches will be enabled for more accurate medicines management [43].

Insights from predictive analytics can already be generated more readily and at a lower cost. To date, predictive models have been used to drive risk scores to identify individuals or sub-populations who are at high risk of non-adherence to medications in populations with conditions such as HIV [44], hypertension [45], inflammatory bowel disease [46] and diabetes [47]. These models can identify high-risk populations for further interventions. For instance, an individual belonging to the high-risk group can be identified as likely to be non-adherent due to a complex medication regimen requiring frequent dosing. To improve adherence, therapies could be personalised such as by changing the medication to reduce the frequency of doses.

Most studies have relied on regression models with data derived from cohort studies that surveyed patient populations on clinical, behavioural and psychosocial variables relevant to medication adherence. Future models will go beyond simple regression techniques with greater deployment of sophisticated data mining techniques such as machine learning. By improving quality, comprehensiveness of data and bringing together large numbers of digitally collected data variables, prediction accuracy will improve.

On the other side, predictive analytics could also be used by business entities, such as pharmaceutical companies, to improve market intelligence. Leveraging on mass data, marketing efforts could be personalised and well-targeted, but such efforts may lead to higher cost to the patient and healthcare system while at the same time promote medications or uses that might not be evidence based. For health insurance companies, it can be argued that a greater integration of data might facilitate more accurate prediction of an individual’s cost profile. However, most jurisdictions, including the US, have moved towards legislating for community-rated insurance with all individuals paying the same premium, thereby reducing the potential for providing insurance only for those at lowest risk.

Although predictive models utilising data from health IT systems are not new, they are mostly proprietary [48, 49]. In reality, such analytics capabilities are not yet part of the routine delivery of care. Health systems are still in the process of learning how to harness their value to improve healthcare quality [50] and medication management.

## Overcoming barriers to eHealth implementation

Despite the potential for cost-savings and added flexibility in improving medicines management, challenges in eHealth implementation must not be disregarded. First, eHealth implementation requires adequate planning and consideration of cost-effectiveness [51]. The failure of the NHS national programme for IT is attributable in large part to inadequate planning. Ambitious plans to scale-up the adoption and interconnectivity of EHRs in the UK backfired when the system was not primed for a rapid and large scale transformation [52]. eHealth interventions for medicines management should be piloted and scaled up progressively. Second, digital literacy is necessary for the adoption of eHealth. Knowledge of technology can improve engagement and receptivity towards technological changes, which is crucial for digital interventions to succeed [53]. Additional training is also required for specific patient sub-groups to improve their digital literacy prior to eHealth interventions in medicines management. Third, patient safety must not be compromised. New technologies may expose users to unintended risks. In a systematic assessment of 46 insulin dose calculator apps, it was found that the majority had problems with the data input function, did not validate numeric inputs and displayed incorrect dose results [54]. Adequate risk management frameworks must be in place to mitigate potential risks posed to patient safety. Finally, health data privacy should be reinforced to increase confidence in eHealth [55]. The widespread acceptance of eHealth in medicines management will occur with time, just like its banking and ecommerce precedents [56], but only when frameworks and secured encrypted networks provide assurance of security.

While the prospects of eHealth are promising, widescale adoption of eHealth interventions at the population level requires further significant research, planning, communication to different population groups and skilled health IT personnel [57, 58].

## Directions for future research

The rapid innovation in eHealth technologies has challenged policymakers and implementers to find a balance between evidence-based integration of technologies and constructive experimentation that could lead to a game-changing breakthrough [59]. eHealth can impact medications management at all three levels: macro (at the level of the healthcare system), meso (clinics, pharmacies) and micro (individual patients and carers). At the micro level, the personalisation of alerts in the way of regular reminders via mobile phone SMS has been found to double the odds of medication adherence [40]. ATCS was also found to be moderately effective at improving the monitoring of health outcomes, which positively influences adherence with medicines. These interventions have high potential for scaling to routine delivery of healthcare. This should be accompanied with an ongoing rigorous evaluation to further improve them (e.g. by further personalisation) and create evidence about long-term effectiveness in real-life. No study of reminders for medication adherence provides data on long-term (over several years) effectiveness, acceptability, etc. Yet, if these interventions are to be embedded in routine delivery of healthcare these answers are critically needed.

EHR, CDSS and CPOE vary in the level of benefits they deliver to health systems in the short term and are challenging to implement at scale. We urgently need more effective strategies for organisational change that ensure adoption, not only by enthusiasts, but by all healthcare professionals alike. We also require rigorous independent head-to-head studies across a wide range of eHealth medicines management and other functionalities of such systems to inform decision-making related to their adoption. This information will be of equal relevance to low-, middle- and high-income countries, and evaluation should allow context-specific interpretation; the same applies to health apps [54, 60, 61].

Greater use of eHealth to improve health literacy at an individual and population level is an obvious priority area for research. There are few, if any, technological barriers and risks are likely to be minimal. For example, simple solutions for electronic provision of patient education materials on medications in low- and middle-income countries where patient education leaflets are not currently provided have the potential to significantly raise the standard of care and positively impact health outcomes. These could be platform-agnostic (i.e. for use on a computer, tablet or smartphone) text, audio or multimedia-rich depending on the context, medication prescribed and patient needs.

The transdisciplinary nature of eHealth calls for a team approach in research that brings multifarious disciplines together with methodological innovations. Traditional randomised controlled trial designs may not be suitable, for example, for evaluation of apps. In many instances, the technology might already be obsolete by the time the results are published, let alone implemented at scale. Too few studies go beyond effectiveness, future eHealth research needs to include and carefully consider, amongst other dimensions, acceptability, human factors and ergonomics, adoption and adaptability, scalability and fidelity of interventions at scale (over time and in different contexts), sustainability, training needs, safety, patient and clinician preferences of different backgrounds, and cost-effectiveness to inform resource allocation decisions at the micro, meso and macro levels.
